# Supplementary material for: Deciphering Staphylococcus aureus–host dynamics using dual activity-based protein profiling of ATP-interacting proteins
Source: mSystems. 2024 Apr 24;9(5):e00179-24. doi: 10.1128/msystems.00179-24 (PMC11097646; doi:10.1128/msystems.00179-24)
Supplement: Supplemental figures — Fig. S1 to S7. [file msystems.00179-24-s0001.pdf]

Supplementary data

Title: Deciphering Staphylococcus aureus-host dynamics using dual Activity-based protein profiling of ATP-interacting proteins .

Authors: Stephen Dela Ahator[1*], Kristin Hegstad[1,2], Christian S. Lentz[1], Mona Johannessen[1*]

Affiliations: [1]Centre for New Antibacterial Strategies (CANS) & Research Group for Host-Microbe Interactions, Department of Medical Biology, Faculty of Health Sciences, UiT- The Arctic University of Norway, 9037 Tromsø. [2] Norwegian National Advisory Unit on Detection of Antimicrobial Resistance, Department of Microbiology and Infection Control, University Hospital of North Norway, Tromsø, Norway

Fig S1

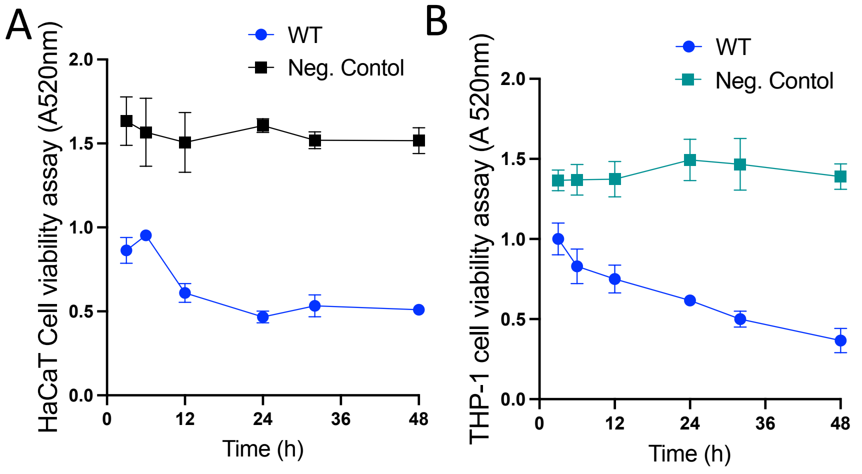

Fig S1:
A. Time-course HaCaT cell and **B**. THP-1 cell viability assay following infection with WT *S. aureus* strain and blank PBS control. Cells were incubated in A. DMEM+ 50µg/ml Gentamicin and B. RPMI 1640+50 50µg/ml Gentamicin. The data displayed are mean ± SD of three independent experiments.

Fig S2

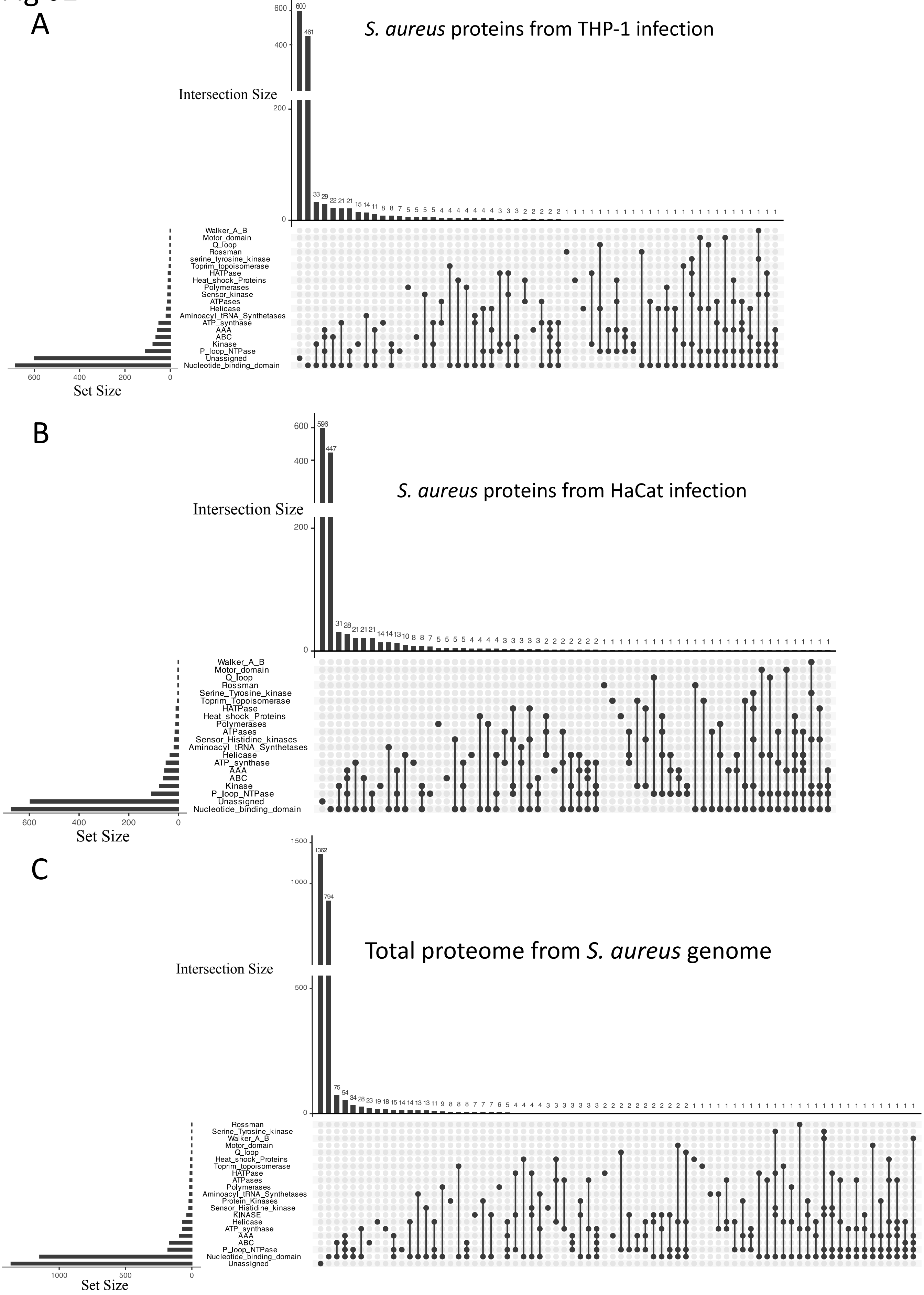

A
S. aureus proteins from THP-1 infection
Intersection Size
Set Size
Walker_A_B
Motor_domain
Q_loop
Rossman
serine_tyrosine_kinase
Toprim_topoisomerase
HATPase
Heat_shock_Proteins
Polymerases
Sensor_kinase
ATPases
Helicase
Aminoacyl_tRNA_Synthetases
ATP_synthase
AAA
ABC
Kinase
P_loop_NTPase
Unassigned
Nucleotide_binding_domain
B
S. aureus proteins from HaCat infection
Intersection Size
Set Size
Walker_A_B
Motor_domain
Q_loop
Rossman
Serine_Tyrosine_kinase
Toprim_Topoisomerase
HATPase
Heat_shock_Proteins
Polymerases
ATPases
Sensor_Histidine_kinases
Aminoacyl_tRNA_Synthetases
Helicase
ATP_synthase
AAA
ABC
Kinase
P_loop_NTPase
Unassigned
Nucleotide_binding_domain
C
Total proteome from S. aureus genome
Intersection Size
Set Size
Rossman
Serine_Tyrosine_kinase
Walker_A_B
Motor_domain
Q_loop
Heat_shock_Proteins
Toprim_topoisomerase
HATPase
ATPases
Polymerases
Aminoacyl_tRNA_Synthetases
Protein_Kinases
Sensor_Histidine_kinase
KINASE
Helicase
ATP_synthase
AAA
ABC
P_loop_NTPase
Nucleotide_binding_domain
Unassigned

Fig S2:

The classification of ATP-probe profiled *S. aureus* proteins following infection in **A**, THP-1 cells and **B**, HaCaT cells. **C**. The classification of proteome in the *S. aureus* USA300 genome using the HMM profile derived from domain search from the UniProt database. After infecting HaCaT and THP-1 cells with bacteria, we discovered that the majority of the resultant proteins which we classified as unassigned, were mostly hypothetical or have undefined domains or metabolic enzymes with domains without prior knowledge of ATP or nucleotide interactions. These unclassified proteins did not correlate with proteins that have annotated ATP-interacting domains, suggesting they may represent a novel class of ATP-related proteins in the bacteria. In comparison, approximately 40-50% of these unassigned proteins were detectable in the *S. aureus* genome using our probes, contrasting with those that have nucleotide-binding domains and do intersect with other known ATP-binding proteins.

Fig S3

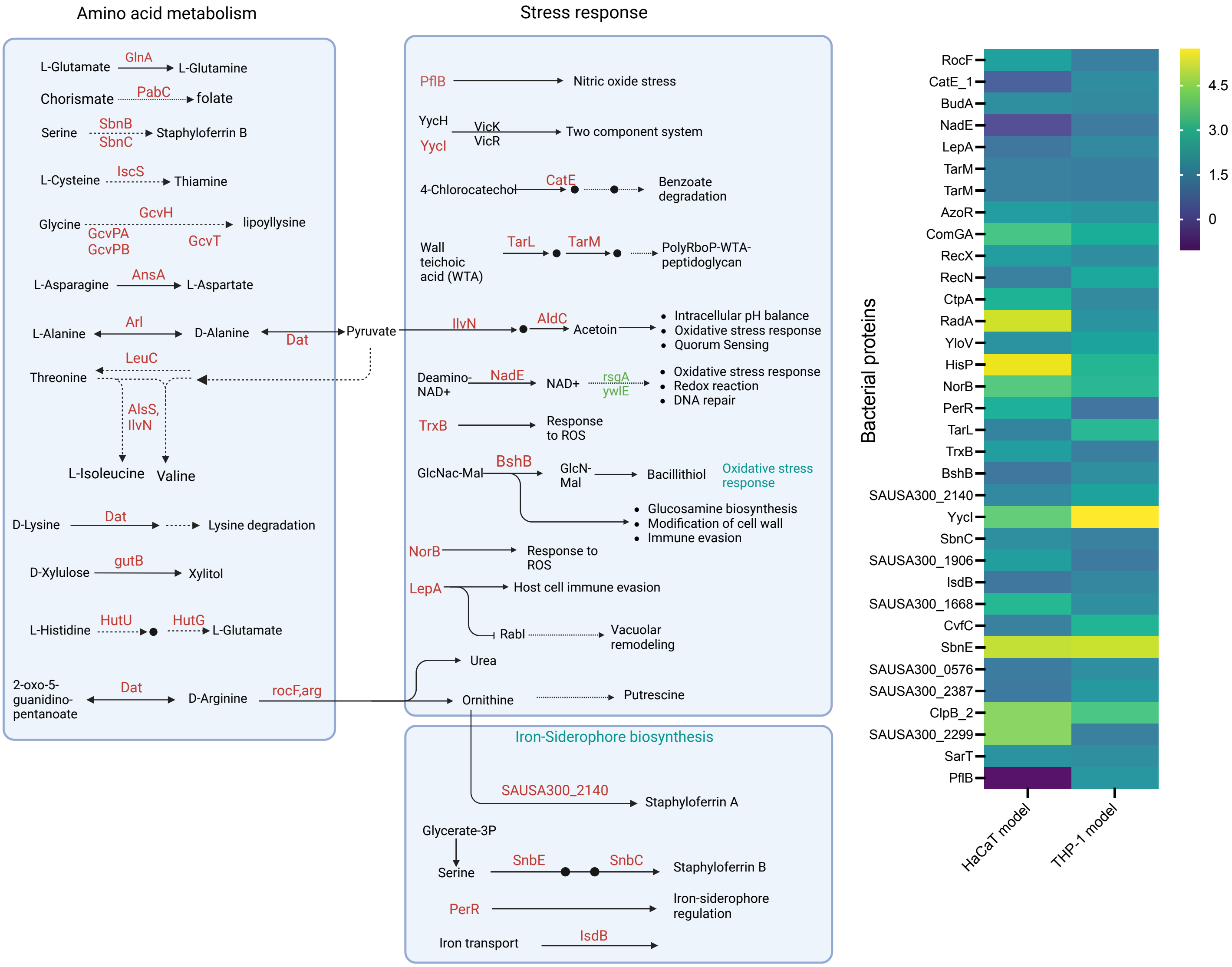

Fig S3:
The heatmap and diagrammatic rendering of the amino acid metabolism and stress response pathway involving bacterial proteins activated and repressed following infection in the THP-1 and HaCaT cells. The heatmap shows the relative activity (log2 value) of the bacterial proteins involved in the amino acid synthesis, metabolism and transport pathway. The metabolic pathway maps are renderings of the pathways derived from the KEGG and EggNog database.

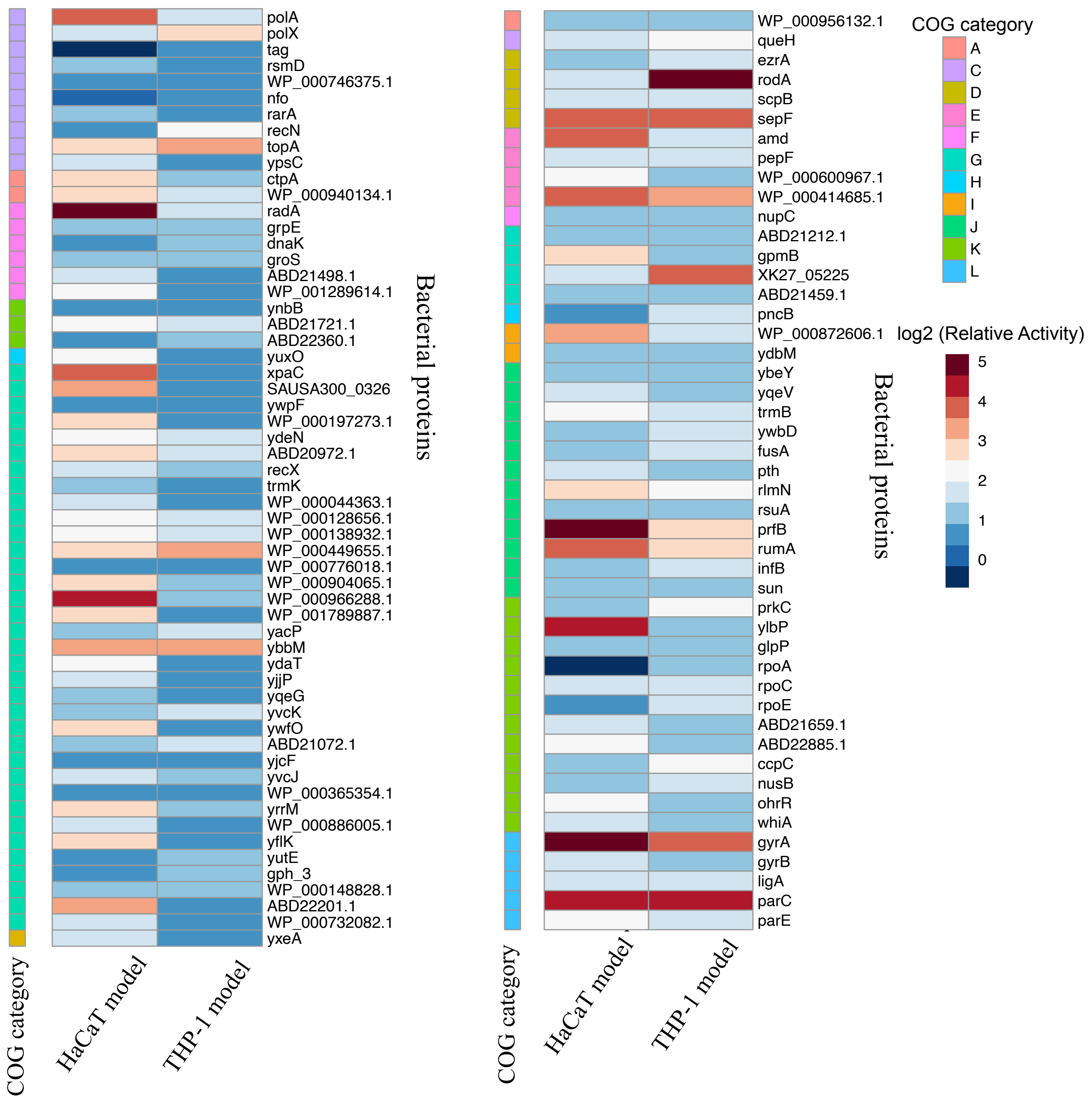

Fig S4:
Heatmap showing the differential activation of *S. aureus* proteins following infection in THP-1 and HaCaT cells. The heatmap scale shows the log2 value for the protein activity.

Fig S5

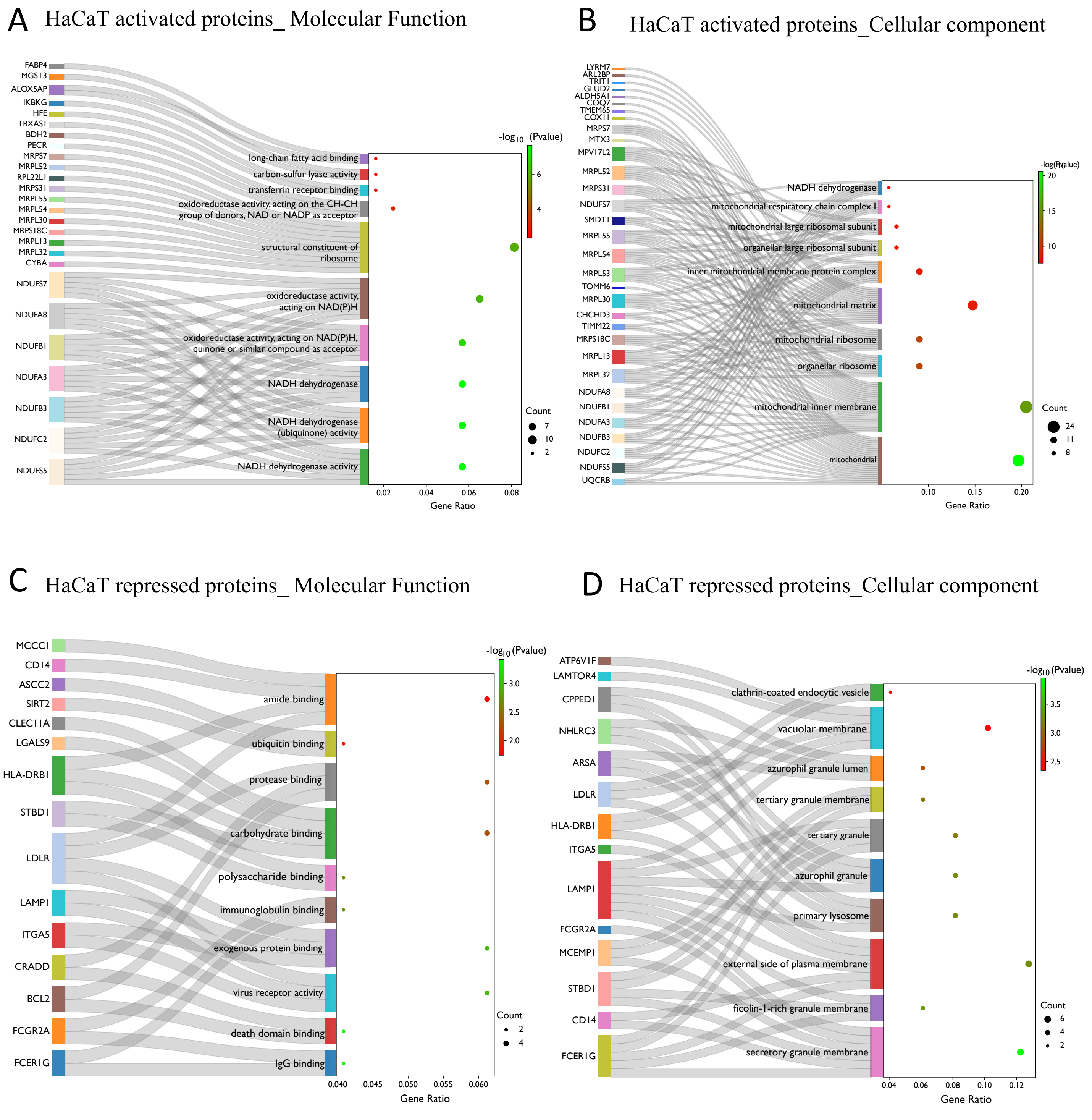

Fig S5:
The categorization of **activated** human proteins from the HaCaT cells enriched in **A**. Molecular function and **B**, Cellular processes following *S. aureus* infection. The categorization of **repressed** human proteins from the HaCaT cells enriched in **C**, Molecular function and **D**, Cellular processes following *S. aureus* infection.

Fig S6

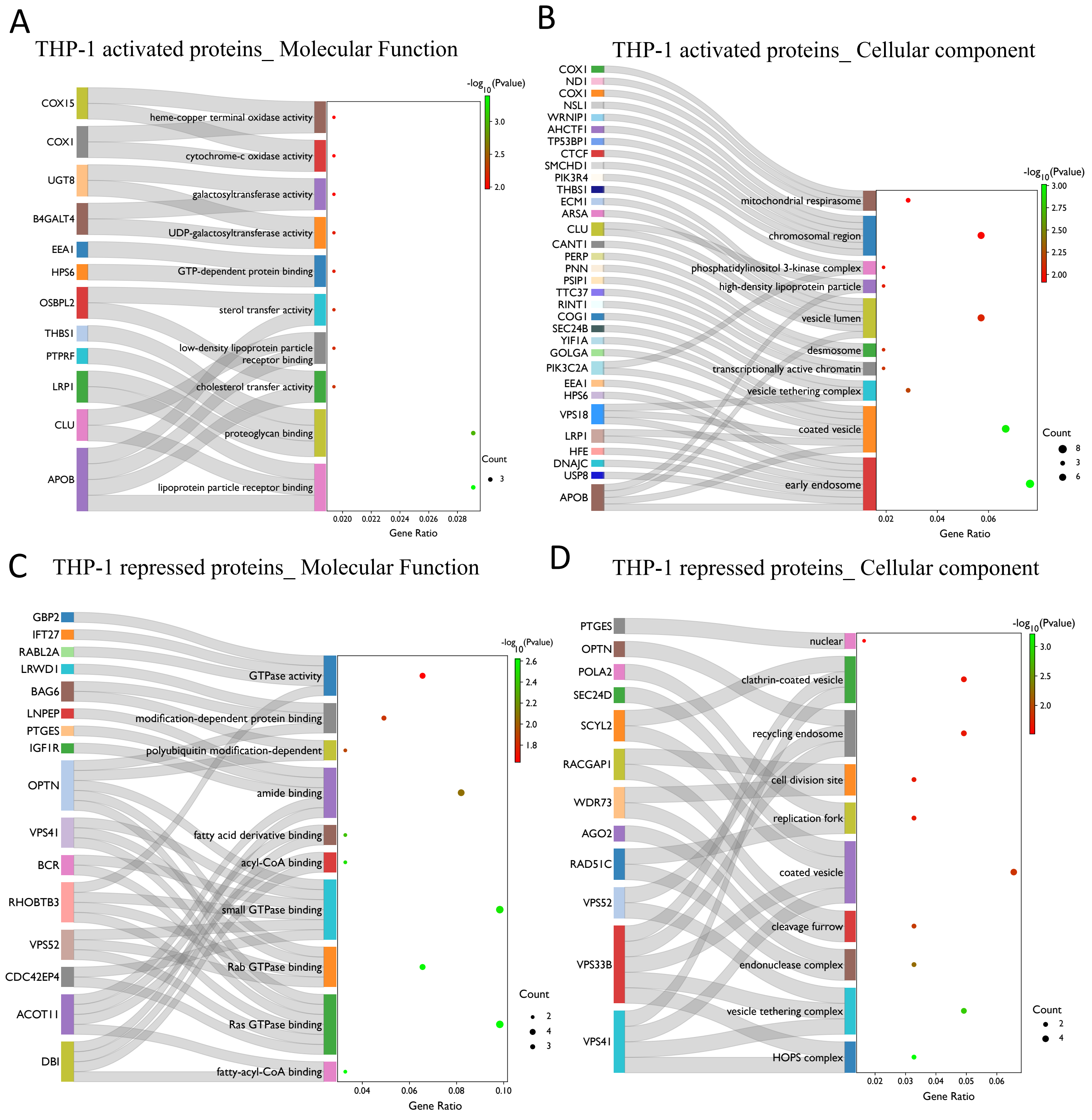

Fig S6:
The categorization of **activated** human proteins from the THP-1 cells enriched in **A**. Molecular function and **B**, Cellular processes following *S. aureus* infection. The categorization of **repressed** human proteins from the THP-1 cells enriched in **C**, Molecular function and **D**, Cellular processes following *S. aureus* infection.

Fig S7

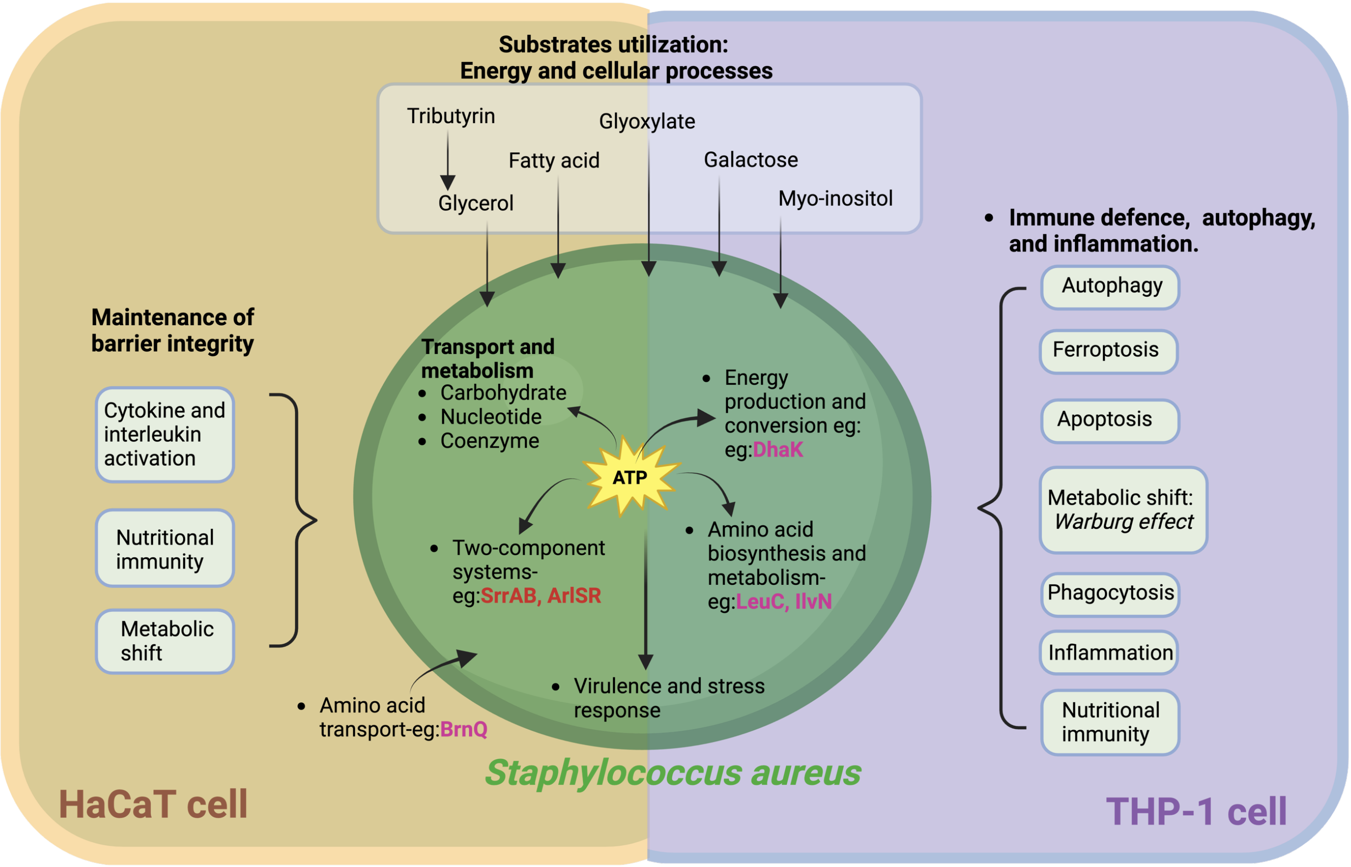

Fig S7. Graphical summary showing the pathways and proteins activated in *S. aureus* and the human cell lines (THP-1 and HaCaT cells) during infection. HaCaT cell and THP-1 cells are shown in orange and purple respectively. *S. aureus* is shown in green.
